# Supplementary material for: Epidemic dynamics of respiratory syncytial virus in current and future climates
Source: Nat Commun. 2019 Dec 4;10:5512. doi: 10.1038/s41467-019-13562-y (PMC6892805; doi:10.1038/s41467-019-13562-y)
Supplement: Supplementary file 1 — Supplementary Information [file 41467_2019_13562_MOESM1_ESM.pdf]

Supplementary Information

Epidemic dynamics of respiratory syncytial virus in current and future climates

Baker et al.

Contents

1 **Supplementary Note 1** 2

2 **Supplementary Figures** 4

3 **Supplementary Tables** 16

1 Supplementary Note 1

While there is no animal model for RSV, laboratory experiments using a guinea pig animal model have revealed humidity and temperature to be important drivers of influenza transmission [4, 5]. Specific humidity, in particular, was found to be correlated with transmission in both laboratory settings [11], and in observational studies [12, 17]. Various hypotheses exist to explain the mechanistic relationship between humidity and transmission [6]. First, it has been suggested that drier conditions increase the duration of suspension of the aerosolized virus particle through evaporation, though there is mixed support for this hypothesis [6, 11]. Second, viral shedding in the nasal passageways has been shown to depend on climate [4]. Finally, virus survival, which may lead to increased transmission at the population level, has been shown to be correlated with specific humidity for both influenza [11], and RSV [16].

Laboratory experiments have not tested for an effect of precipitation on influenza transmission, however observational studies for both influenza and RSV have recognized the correlation between precipitation and incidence in tropical locations [7, 13, 8, 15, 14].

The saturation vapor pressure e_s is related to temperature T roughly exponentially through the Clausius-Clapeyron relationship

$$\frac{\Delta e_s}{e_s} \backsimeq \frac{L_v M_w}{1000R^*}\frac{\Delta T}{T^2} \tag{1}$$

where L_v and M_w are the latent heat of vaporization and molecular weight of water respectively, and R^* is the universal gas constant.

Given that for the range of temperatures observed in the earth's atmosphere the total pressure p is much larger than e_s, the specific humidity q can be related to the saturation vapor pressure through the expression

$$q \backsimeq \frac{0.622RH}{p} e_s \tag{2}$$

where RH is the relative humidity.

One long held assumption in climate modeling is the relative stability of the distribution of relative humidity over time. This has been supported by physical arguments regarding the continuity of the distribution of land and water [2] and the constraint of moisture condensing out of supersaturated air that broadly accounts for the humidity of tropospheric air parcels above the boundary layer[1], as well as by general circulation models with high vertical

resolution [3]. It follows then that q should change by approximately the same amount per degree K as e_s [10], and that as surface temperatures rise so should tropospheric specific humidity [1].

Under atmospheric conditions the August-Roche-Magnus forumula provides a good approximation for the relationship between e_s and T (in Celsius)

$$e_s = 6.1094 \exp(\frac{17.625T}{T + 243.04}) \quad (3)$$

The equation implies an exponential dependence of q on T, i.e.

$$q \propto \mathrm{e}^T \quad (4)$$

Supplementary Fig. 10 shows the estimated effect of temperature on log transmission. The result follows a shallower gradient than the specific humidity plot Supplementary Fig. 8, consistent with a logarithmic relationship.

2 Supplementary Figures

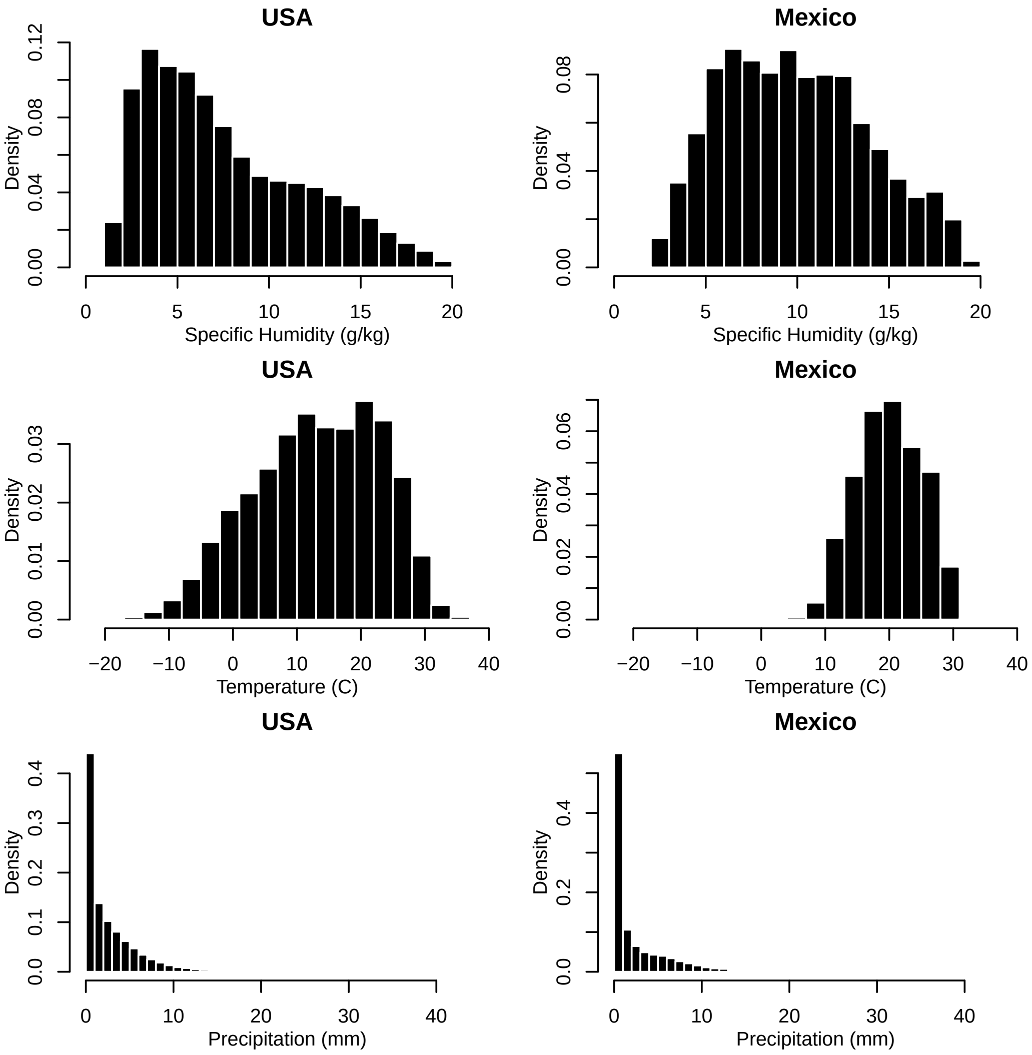

Figure 1: Histograms of weekly specific humidity, temperature and precipiation distributions for the locations in the dataset.

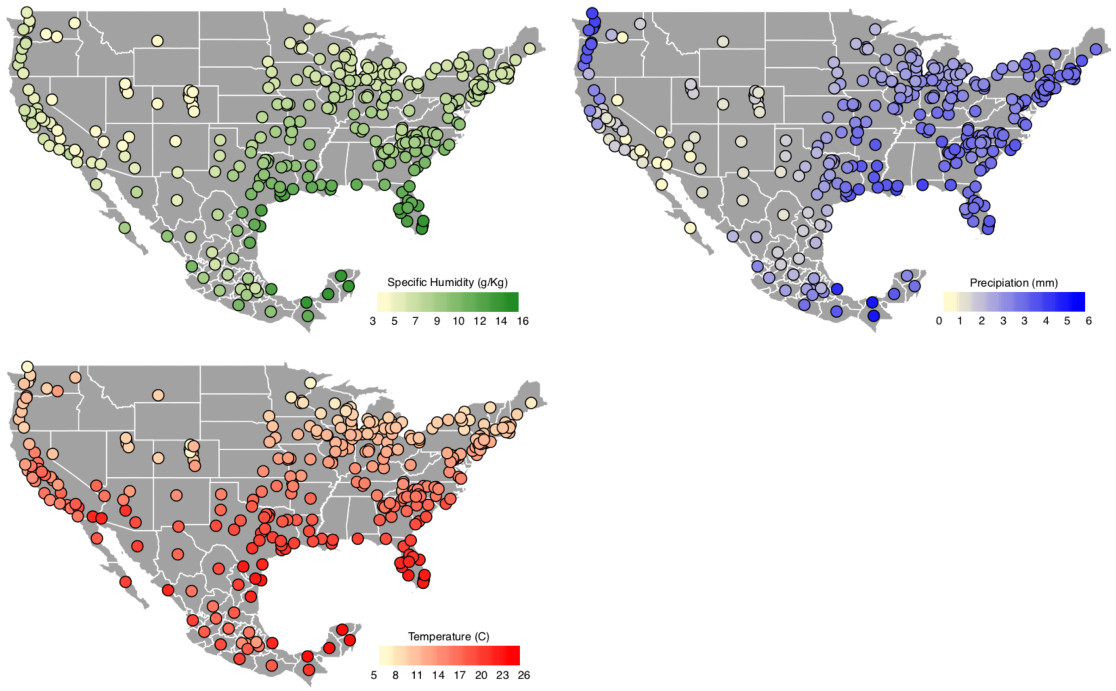

Figure 2: Maps of average weekly humidity, precipitation, temperature for all locations in the dataset.

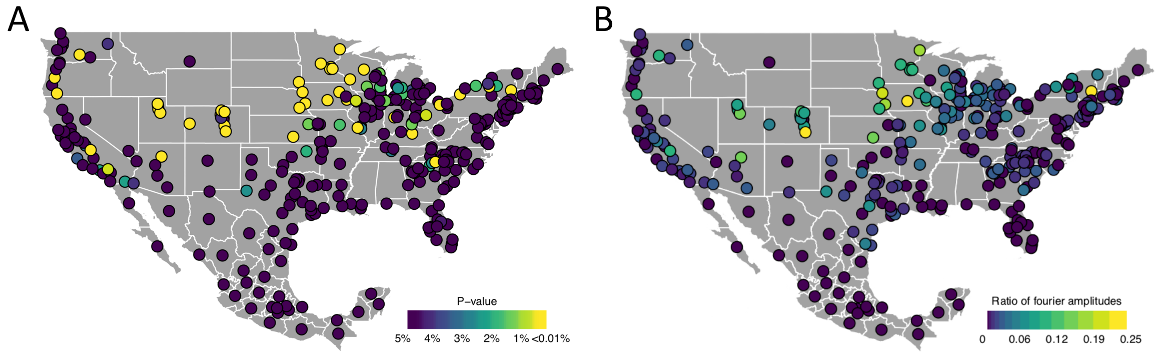

Figure 3: Two measures of the biennial signal: A) shows the significance of the biennial signal based on the Lomb-Scargle periodogram. B) shows the ratio of biennial to annual fourier amplitudes, used in [9].

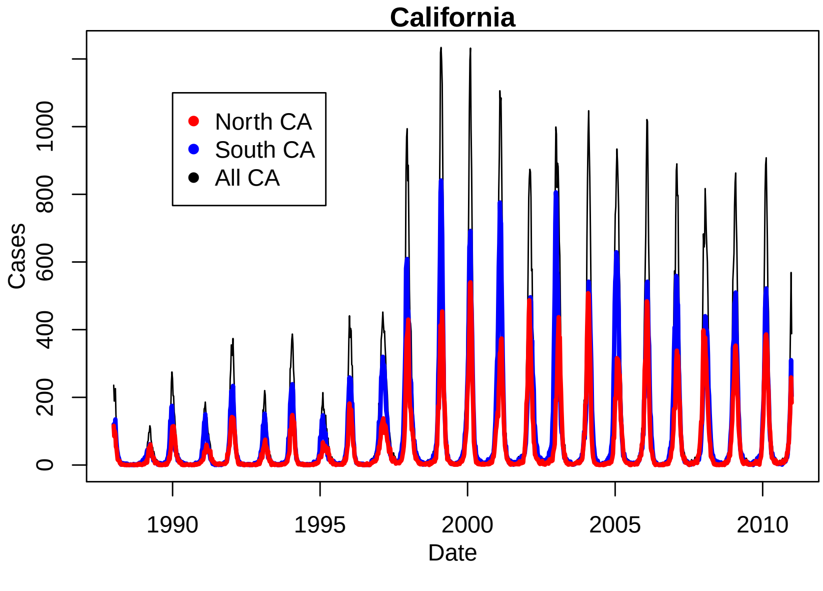

Figure 4: Total weekly incidence summed over northern and southern Californian counties, as well as for the whole state. Biennial patterns are observable at the sub-state level.

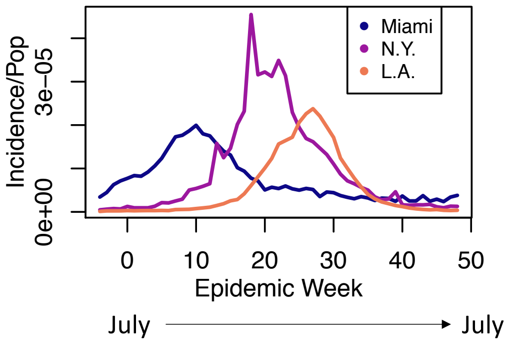

Figure 5: Average incidence per population for Miami, New York and Los Angeles plotted against epidemic week, defined as the first week in July.

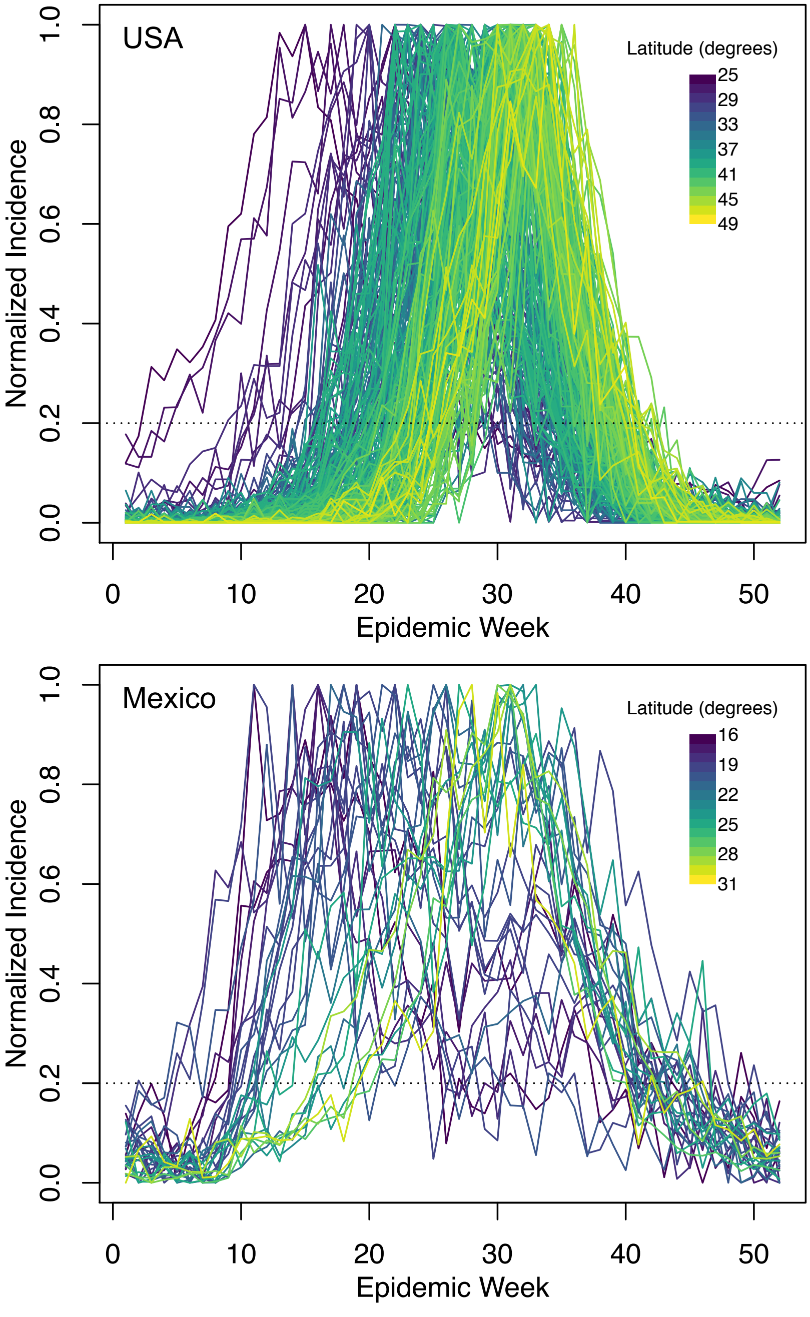

Figure 6: Normalized incidence in the USA and Mexico, plotted against epidemic week (first week of July). Color gradient represents latitude. Dashed line represents the threshold value (0.2) to define the epidemic onset.

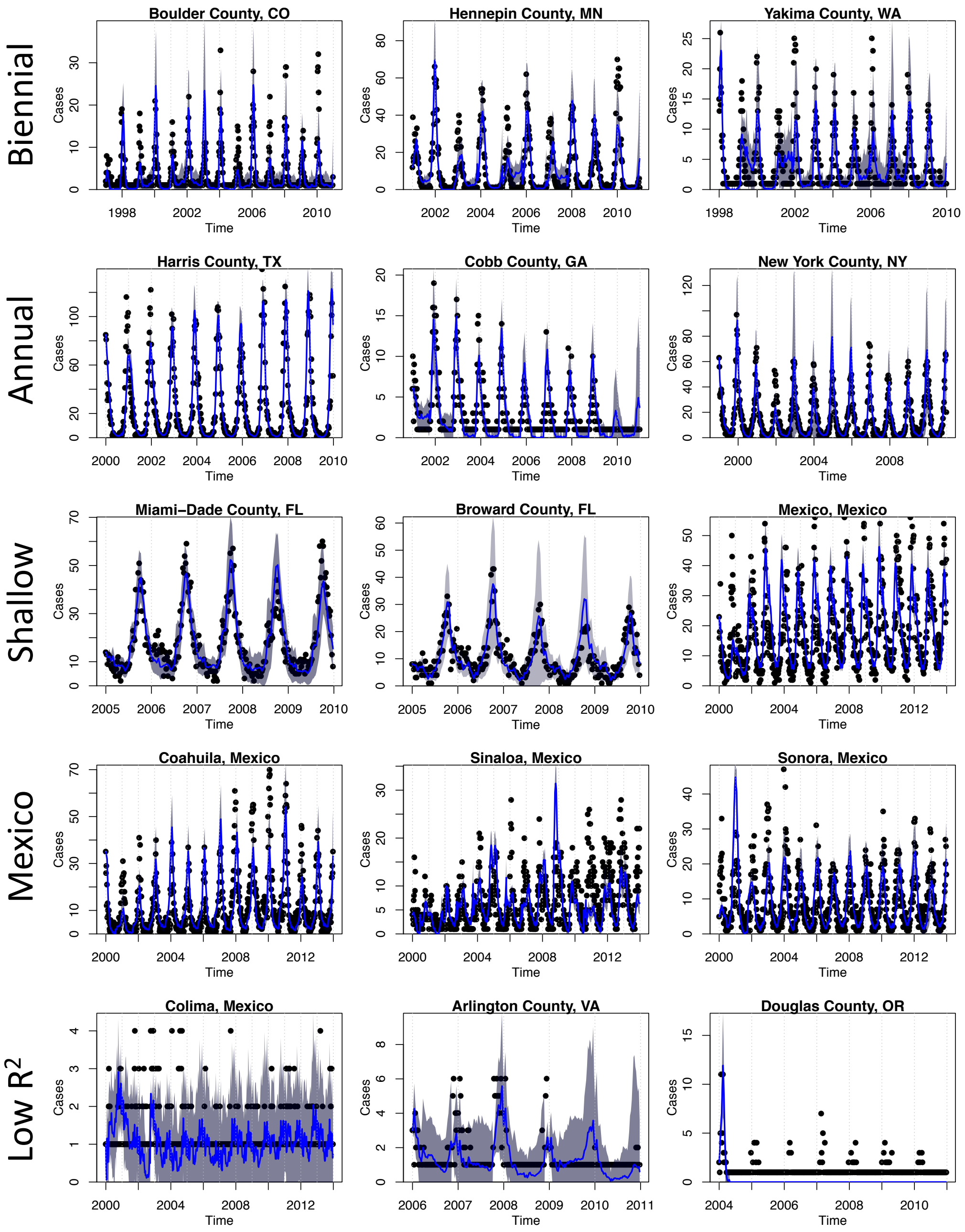

Figure 7: Mean TSIR model fit (blue) with confidence intervals (grey) plotted alongside data (black). The model is able to captue the biennial, annual and shallow trough dynamics. Poor models fits (last row) tend to occur in locations with very low case numbers.

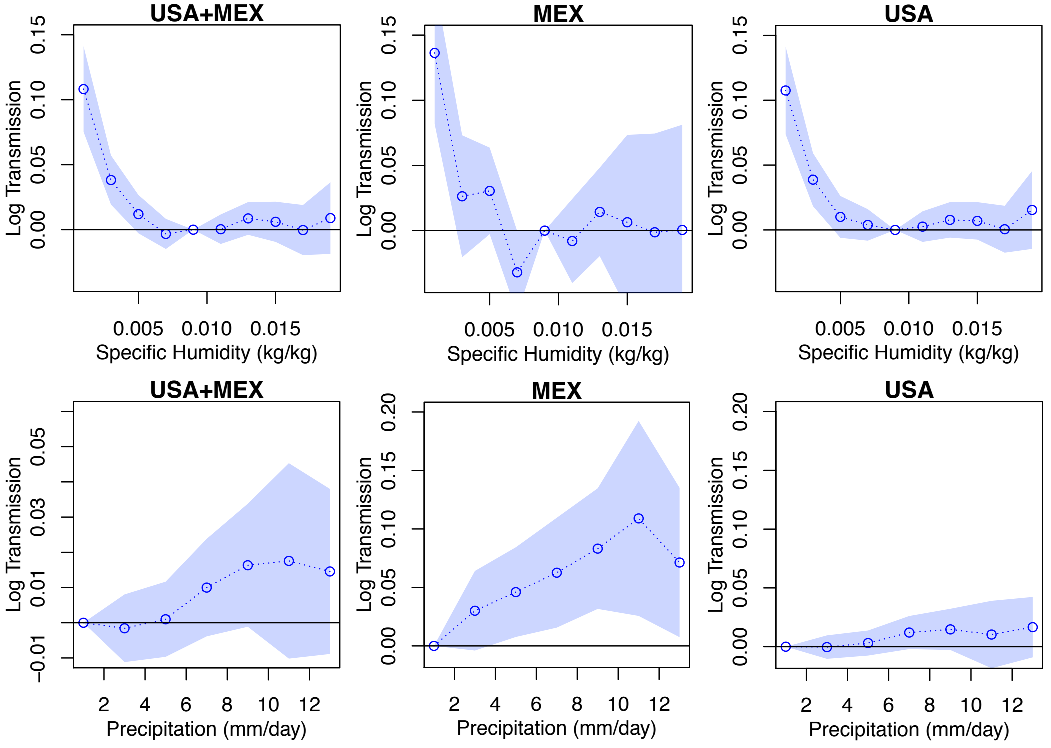

Figure 8: Results from the regression model in equation 6, showing the effect of climate on log transmission, using a flexible binned model to reveal the functional form of the humidity and precipitation relationships. 95% confidence intervals are shown.

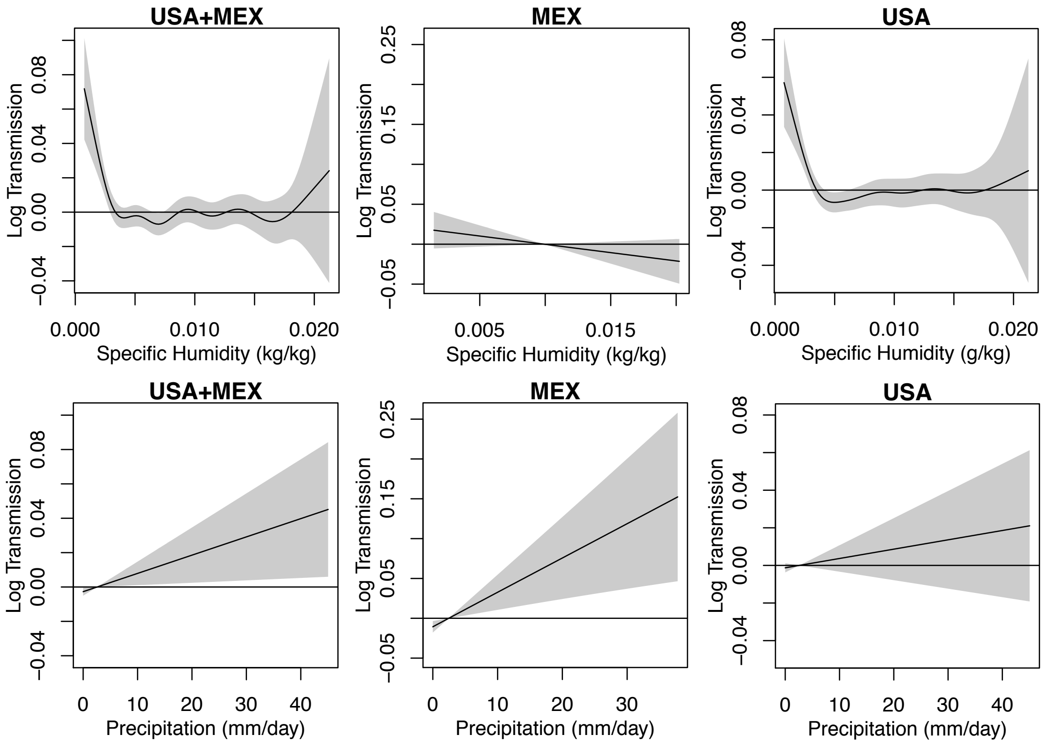

Figure 9: Results from using a general additive model (GAM) to find the effect of climate on transmission. The GAM model was fit in two stages: fixed effects were fitted first using the regression model and then the GAM was fitted to the residuals of the regression.

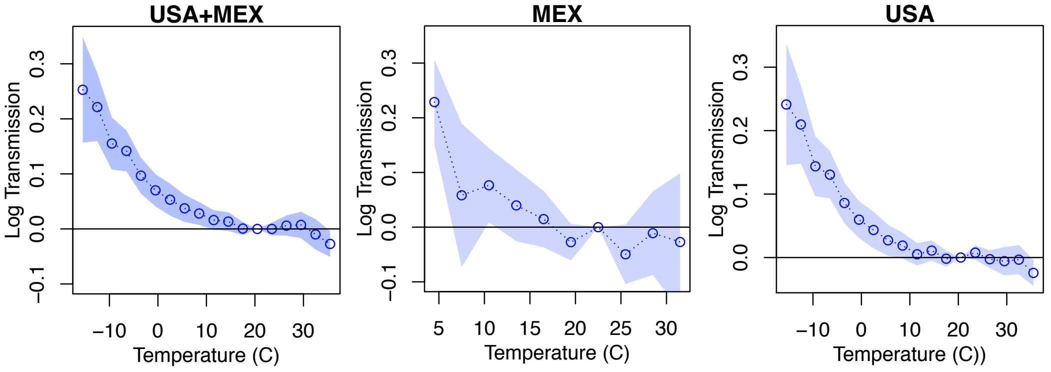

Figure 10: The effect of temperature on transmission using the binned model.

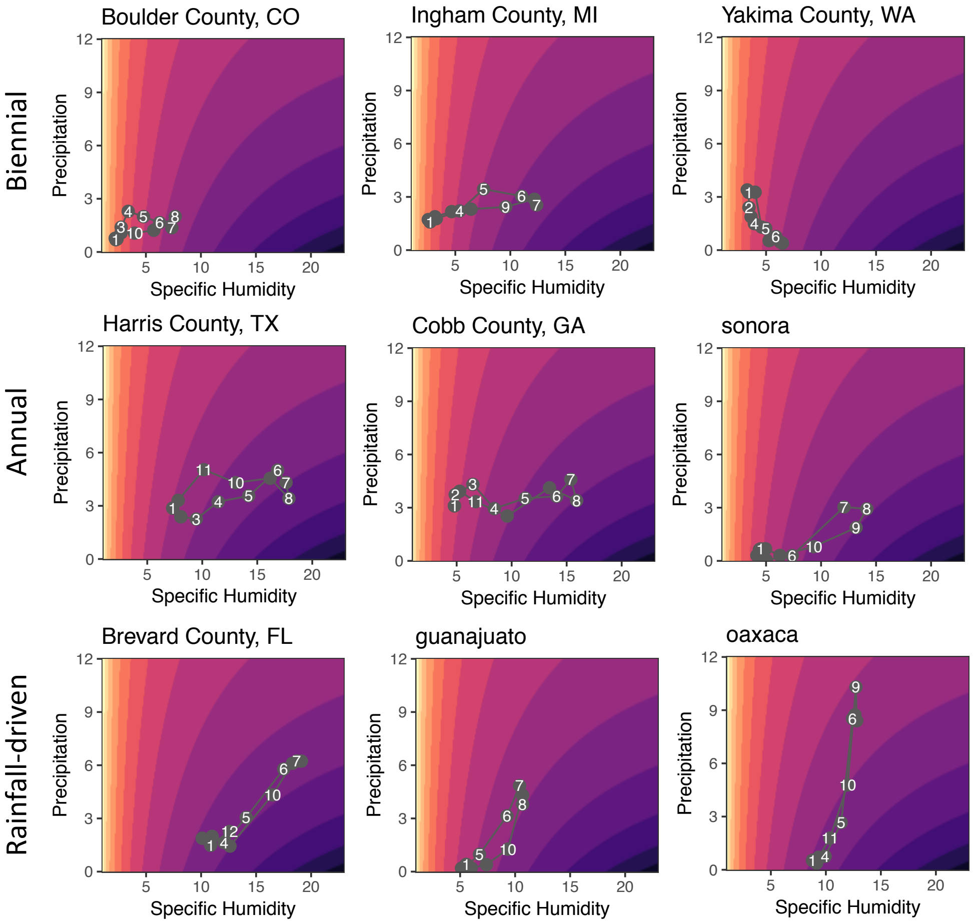

Figure 11: Seasonal trajectories (grey points with month-of-year) for nine locations in the dataset against predicted transmission (surface plot). Biennial locations tend to have high predicted transmission in the winter months. Annual locations have lower predicted transmission on average. Rainfall-driven locations experience intra-annual variability in precipitation that trades off against humidity effects.

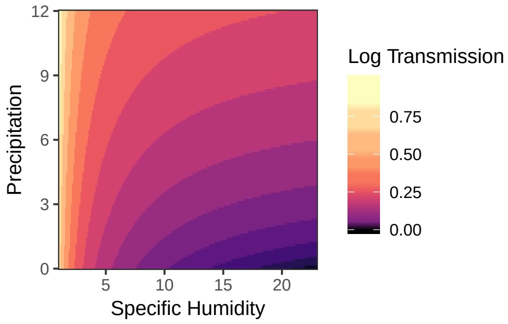

Figure 12: The predicted effect of specific humidity and precipitation on transmission removing fixed effects (equation 7). This allows for specific humidity and precipitation to fully drive variability in transmission though the effect may be biased by other seasonally varying factors. Predicted effects are larger than previously fitted results and are significant ($p<<0.001$). The relative effect for rainfall is greater than in the previous model, though evidence from Mexico suggests our pooled dataset result for precipitation is biased downwards.

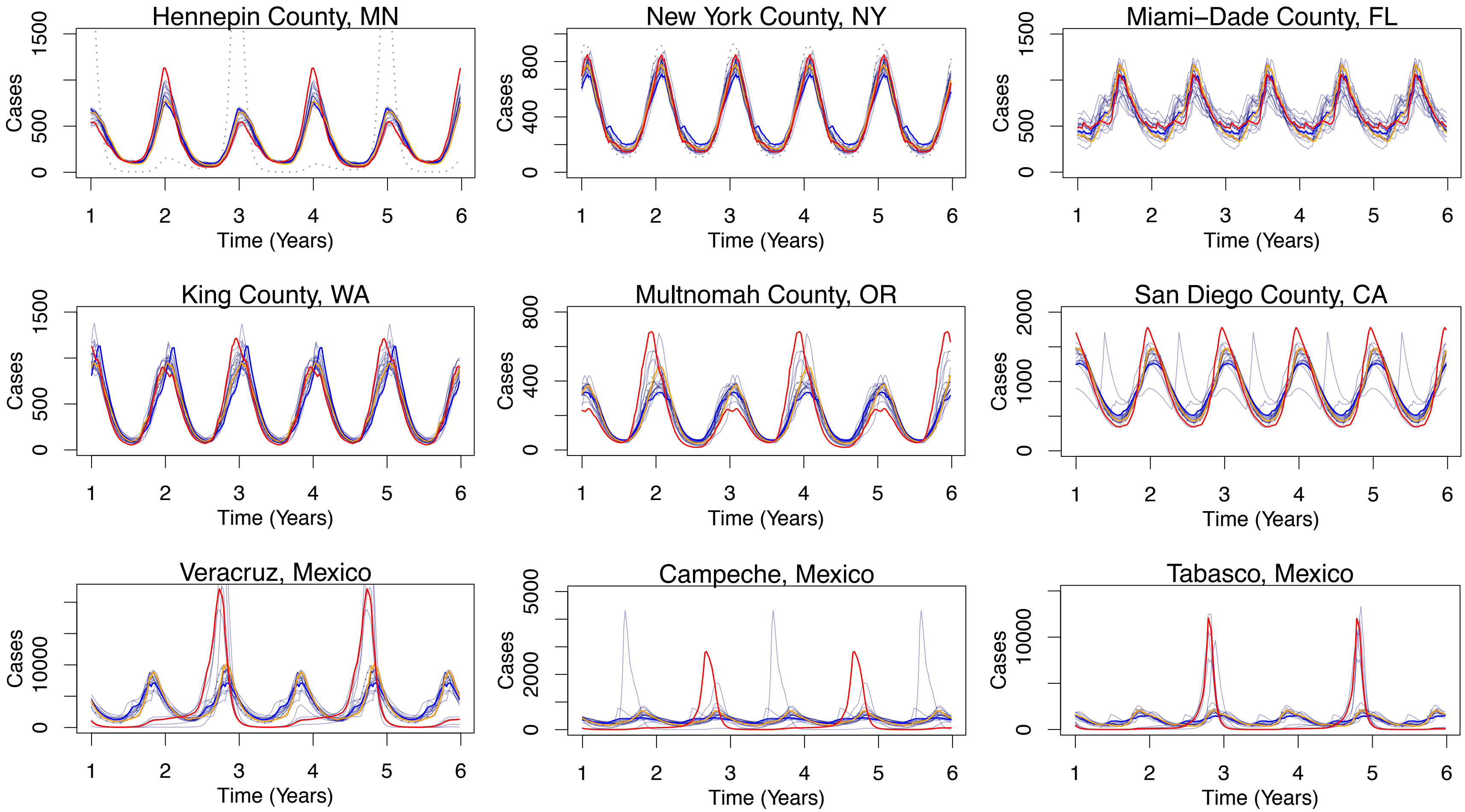

Figure 13: Simulations of future incidence for nine locations, the top row are locations from Fig. 2, the middle row are US west coast locations where rainfall projections have differing effects on dynamics, the last row are three more locations in Mexico. Each line within the plot represents the RSV trajectory based on precipitation projections from an individual climate model where red line is the upper 90th percentile in terms of predicted swing in transmission, orange is 50th percentile and blue in the 10th percentile. Projections for specific humidity are held at the multi-model mean. The dashed line, where visible, is the baseline scenario without climate change.

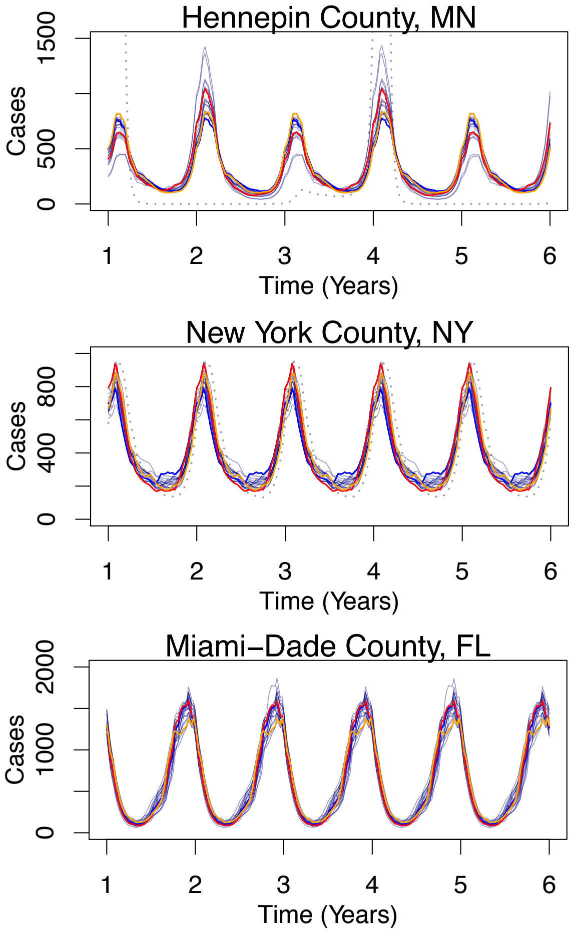

Figure 14: Simulations of future incidence for three locations in S12. These simulations are based on a regression model that includes month fixed effects, representing consistent timing of schooling. As above, each line within the plot represents the RSV trajectory based on precipitation projections from an individual climate model where red line is the upper 90th percentile in terms of predicted swing in transmission, orange is 50th percentile and blue in the 10th percentile. Projections for specific humidity are held at the multi-model mean. The dashed line, where visible, is the baseline scenario without climate change.

3 Supplementary Tables

	Dependent variable:					
	Onset Week					
	(BOTH)	(BOTH)	(BOTH)	(MEX)	(MEX)	(MEX)
Spec Hum (g/kg)	-1.028^{***}			0.541		
	(0.226)			(0.880)		
Precip (mm)		-0.530^{***}			0.807	
		(0.152)			(0.636)	
Temperature (C)			-0.318^{***}			-0.906^{*}
			(0.108)			(0.521)
Observations	3,263	3,263	3,263	384	384	384
Adjusted R^2	0.651	0.650	0.650	0.388	0.389	0.390
Note:						*p<0.1; **p<0.05; ***p<0.01

Table 1: Regression of climate variables on onset week. The model includes location fixed effect to control for spatial heterogeneities in onset determinants. The first three models use the combined USA and Mexico sample, the final three models use Mexico only. Standard errors are shown in parentheses.

	Dependent variable:		
	Log Transmission		
	USA + MEX	USA	MEX
1/Specific Humidity	2.415e-04***	2.414e-04***	1.378e-04
	(2.665e-05)	(2.724e-05)	(1.150e-04)
Precipitation	1.853e-03***	1.033e-03**	7.427e-03***
	(5.095e-04)	(4.829e-04)	(1.868e-03)
Observations	119,802	97,992	21,810
R^2	0.825	0.819	0.469
Adjusted R^2	0.818	0.812	0.451
Note:			*p<0.1; **p<0.05; ***p<0.01

Table 2: The results from regression model (6), reported in Fig. 2A in the paper. Standard errors are shown in parentheses.

	Dependent variable:		
	Log Transmission		
	USA + MEX	USA	MEX
1/Specific Humidity	2.110e-04***	2.046e-04***	1.449-04
	(3.333e-05)	(2.982e-05)	(1.280-04)
Precipitation	1.651e-03*	2.564e-04	8.527e-03***
	(8.498e-04)	(9.901e-04)	(2.154-03)
Observations	84,573	66,514	18,059
R^2	0.755	0.724	0.446
Adjusted R^2	0.735	0.698	0.422
Note:	*p<0.1; **p<0.05; ***p<0.01		

Table 3: Robustness check. The results from regression model (6) where the transmission rate, β, is not calculated if I_{t+1} or I_t is zero. In the main specification we add 1 to zero observations. Standard errors are shown in parentheses.

	Dependent variable:		
	Log Transmission		
	(White)	(Weighted)	(Clustered)
1/Specific Humidity	2.415e-04***	3.070e-04***	2.415e-04 ***
	(3.297e-05)	(2.534e-05)	(2.665e-05)
Precipitation	1.853e-03***	1.608e-03***	1.853e-03***
	(3.950e-04)	(4.811e-04)	(5.095e-04)
Observations	119,802	119,802	119,802
R^2	0.825	0.811	0.825
Adjusted R^2	0.818	0.804	0.818
Note:			*p<0.1; **p<0.05; ***p<0.01

Table 4: Robustness check. A comparison of the main results using different standard error corrections to account for heteroskedasticity. We use White's standard errors, weighted standard errors and location-clustered standard errors. Standard errors are shown in parantheses.

	Dependent variable:		
	Log Transmission		
	USA + MEX	USA	MEX
1/Specific Humidity	1.809e-04***	1.787e-04***	1.378e-04
	(2.143e-05)	(2.174e-05)	(1.150e-04)
Precipitation	1.002e-03**	4.383e-04	7.427e-03***
	(4.082e-04)	(3.932e-04)	(1.868e-03)
Observations	184,898	163,088	21,810
R^2	0.828	0.825	0.469
Adjusted R^2	0.821	0.817	0.451
Note:			*p<0.1; **p<0.05; ***p<0.01

Table 5: Robustness check. The main results from equation 6 where we do not remove data from locations with poor TSIR fit ($R^2 < 0.5$) . In the main specification we remove data from these locations. Standard errors are shown in parentheses.

	Dependent variable:		
	Log Transmission		
	(USA + MEX)	(USA)	(MEX)
1/Specific Humidity	2.050e-04***	2.020e-04***	1.378e-04
	(3.317e-05)	(3.426e-05)	(1.150e-04)
Precipitation	1.526e-03***	4.630e-04	7.427-03***
	(5.862e-04)	(5.557e-04)	(1.868e-03)
Observations	104,187	82,377	21,810
R^2	0.819	0.824	0.469
Adjusted R^2	0.812	0.817	0.451
Note:			*p<0.1; **p<0.05; ***p<0.01

Table 6: Robustness check. The main results from equation 6 where we remove observations from location with an average population of less than 500,000. Standard errors are shown in parentheses.

	Dependent variable:		
	Log Transmission		
	(USA + MEX)	(USA)	(MEX)
1/Specific Humidity	1.886e-04***	1.861e-04***	1.391e-04
	(2.381e-05)	(2.424e-05)	(1.115e-04)
Precipitation	1.000e-03**	3.174e-04	7.427e-03***
	(4.642e-04)	(4.481e-04)	(1.8694e-03)
Observations	184,898	163,088	21,810
R^2	0.778	0.809	0.404
Adjusted R^2	0.769	0.802	0.383
Note:			*p<0.1; **p<0.05; ***p<0.01

Table 7: Robustness check. The main results from equation 6 where we use a negative binomial model to fit the TSIR. For comparison, see Supplementary Table 5. Standard errors are shown in parentheses.

	Dependent variable:	
	Log Transmission	
	(USA + MEX)	(USA)
1/Specific Humidity	1.615e-04***	1.590e-04***
	(2.132e-05)	(2.162e-052)
Precipitation	1.290e-03**	7.520e-04
	(5.128e-04)	(5.204e-04)
Observations	184,847	163,037
R^2	0.730	0.724
Adjusted R^2	0.720	0.713
Note:	*p<0.1; **p<0.05; ***p<0.01	

Table 8: Robustness check. We use bronchiolitis hospitalization data from the USA instead of RSV hospitalization data to check whether bronchiolitis reveals similar results for RSV and therefore is worthwhile proxy in Mexico. The regression model was run on the whole sample and can be directly compared to the previous Supplementary Table 5. We find a similar effect size for both variables. In the USA sample, bronchiolitis data has a slightly weaker relationship with specific humidity than in the RSV model, suggesting some noise is introduced by using bronchiolitis as a proxy for RSV. Standard errors are shown in parentheses.

	Dependent variable:
	Log Transmission
1/Specific Humidity	2.588e-04***
	(3.235e-05)
Lag 1/Specific Humidity	-5.382e-05
	(3.650e-05)
Precipitation	1.899e-03***
	(5.055e-04)
Lag Precipitation	4.940e-04
	(5.359e-04)
Observations	119,802
R^2	0.825
Adjusted R^2	0.818
Note:	*p<0.1; **p<0.05; ***p<0.01

Table 9: Robustness check. The effect of lagged (by one week) specific humidity and lagged precipitation on transmission. Lagged variables do not affect transmission. Standard errors are shown in parentheses.

Supplementary References

[1] Myles R Allen and William J Ingram. Constraints on future changes in climate and the hydrologic cycle. *Nature*, 419(6903):224, 2002.

[2] Svante Arrhenius. Xxxi. on the influence of carbonic acid in the air upon the temperature of the ground. *The London, Edinburgh, and Dublin Philosophical Magazine and Journal of Science*, 41(251):237–276, 1896.

[3] WJ Ingram. On the robustness of the water vapor feedback: Gcm vertical resolution and formulation. *Journal of Climate*, 15(9):917–921, 2002.

[4] Anice C Lowen, Samira Mubareka, John Steel, and Peter Palese. Influenza virus transmission is dependent on relative humidity and temperature. *PLoS pathogens*, 3(10):e151, 2007.

[5] Anice C Lowen and John Steel. Roles of humidity and temperature in shaping influenza seasonality. *Journal of virology*, 88(14):7692–7695, 2014.

[6] Linsey C Marr, Julian W Tang, Jennifer Van Mullekom, and Seema S Lakdawala. Mechanistic insights into the effect of humidity on airborne influenza virus survival, transmission and incidence. *Journal of the Royal Society Interface*, 16(150):20180298, 2019.

[7] Pablo Obando-Pacheco, Antonio José Justicia-Grande, Irene Rivero-Calle, Carmen Rodríguez-Tenreiro, Peter Sly, Octavio Ramilo, Asunción Mejías, Eugenio Baraldi, Nikolaos G Papadopoulos, Harish Nair, et al. Respiratory syncytial virus seasonality: a global overview. *The Journal of infectious diseases*, 217(9):1356–1364, 2018.

[8] SB Omer, A Sutanto, H Sarwo, M Linehan, IGG Djelantik, D Mercer, V Moniaga, LH Moulton, A Widjaya, P Muljati, et al. Climatic, temporal, and geographic characteristics of respiratory syncytial virus disease in a tropical island population. *Epidemiology & Infection*, 136(10):1319–1327, 2008.

[9] Virginia E Pitzer, Cecile Viboud, Wladimir J Alonso, Tanya Wilcox, C Jessica Metcalf, Claudia A Steiner, Amber K Haynes, and Bryan T Grenfell. Environmental drivers of the spatiotemporal dynamics of respiratory syncytial virus in the united states. *PLoS pathogens*, 11(1):e1004591, 2015.

[10] Brian EJ Rose and M Cameron Rencurrel. The vertical structure of tropospheric water vapor: Comparing radiative and ocean-driven climate changes. *Journal of Climate*, 29(11):4251–4268, 2016.

[11] Jeffrey Shaman and Melvin Kohn. Absolute humidity modulates influenza survival, transmission, and seasonality. *Proceedings of the National Academy of Sciences*, 106(9):3243–3248, 2009.

[12] Jeffrey Shaman, Virginia E Pitzer, Cécile Viboud, Bryan T Grenfell, and Marc Lipsitch. Absolute humidity and the seasonal onset of influenza in the continental united states. *PLoS biology*, 8(2):e1000316, 2010.

[13] Lynette Pei-Chi Shek and Bee-Wah Lee. Epidemiology and seasonality of respiratory tract virus infections in the tropics. *Paediatric respiratory reviews*, 4(2):105–111, 2003.

[14] James Tamerius, Martha I Nelson, Steven Z Zhou, Cécile Viboud, Mark A Miller, and Wladimir J Alonso. Global influenza seasonality: reconciling patterns across temperate and tropical regions. *Environmental health perspectives*, 119(4):439–445, 2010.

[15] James D Tamerius, Jeffrey Shaman, Wladmir J Alonso, Kimberly Bloom-Feshbach, Christopher K Uejio, Andrew Comrie, and Cécile Viboud. Environmental predictors of seasonal influenza epidemics across temperate and tropical climates. *PLoS pathogens*, 9(3):e1003194, 2013.

[16] Julian W Tang. The effect of environmental parameters on the survival of airborne infectious agents. *Journal of the Royal Society Interface*, 6(suppl_6):S737–S746, 2009.

[17] Dennis E te Beest, Michiel van Boven, Mariëtte Hooiveld, Carline van den Dool, and Jacco Wallinga. Driving factors of influenza transmission in the netherlands. *American journal of epidemiology*, 178(9):1469–1477, 2013.
