# Supplementary material for: Epidemic dynamics of respiratory syncytial virus in current and future climates
Source: Nat Commun. 2019 Dec 4;10:5512. doi: 10.1038/s41467-019-13562-y (PMC6892805; doi:10.1038/s41467-019-13562-y)
Supplement: Supplementary file 3 — Reporting Summary [file 41467_2019_13562_MOESM3_ESM.pdf]

nature research

Corresponding author(s): Rachel E. Baker

Last updated by author(s): Nov 5, 2019

# Reporting Summary

Nature Research wishes to improve the reproducibility of the work that we publish. This form provides structure for consistency and transparency in reporting. For further information on Nature Research policies, see Authors & Referees and the Editorial Policy Checklist .

## Statistics

For all statistical analyses, confirm that the following items are present in the figure legend, table legend, main text, or Methods section.

| n/a | Confirmed | |
|---|---|---|
| ☐ | ☒ | The exact sample size (*n*) for each experimental group/condition, given as a discrete number and unit of measurement |
| ☐ | ☒ | A statement on whether measurements were taken from distinct samples or whether the same sample was measured repeatedly |
| ☐ | ☒ | The statistical test(s) used AND whether they are one- or two-sided<br>*Only common tests should be described solely by name; describe more complex techniques in the Methods section.* |
| ☐ | ☒ | A description of all covariates tested |
| ☐ | ☒ | A description of any assumptions or corrections, such as tests of normality and adjustment for multiple comparisons |
| ☐ | ☒ | A full description of the statistical parameters including central tendency (e.g. means) or other basic estimates (e.g. regression coefficient) AND variation (e.g. standard deviation) or associated estimates of uncertainty (e.g. confidence intervals) |
| ☐ | ☒ | For null hypothesis testing, the test statistic (e.g. *F*, *t*, *r*) with confidence intervals, effect sizes, degrees of freedom and *P* value noted<br>*Give P values as exact values whenever suitable.* |
| ☒ | ☐ | For Bayesian analysis, information on the choice of priors and Markov chain Monte Carlo settings |
| ☒ | ☐ | For hierarchical and complex designs, identification of the appropriate level for tests and full reporting of outcomes |
| ☐ | ☒ | Estimates of effect sizes (e.g. Cohen's *d*, Pearson's *r*), indicating how they were calculated |

*Our web collection on statistics for biologists contains articles on many of the points above.*

## Software and code

Policy information about availability of computer code

| | |
|---|---|
| Data collection | No software was used to collect data in this study. |
| Data analysis | Data analysis was performed using R, with model fitting using the tsiR package (https://cran.r-project.org/web/packages/tsiR/tsiR.pdf). |

For manuscripts utilizing custom algorithms or software that are central to the research but not yet described in published literature, software must be made available to editors/reviewers. We strongly encourage code deposition in a community repository (e.g. GitHub). See the Nature Research guidelines for submitting code & software for further information.

## Data

Policy information about availability of data

All manuscripts must include a data availability statement. This statement should provide the following information, where applicable:
- Accession codes, unique identifiers, or web links for publicly available datasets
- A list of figures that have associated raw data
- A description of any restrictions on data availability

The Mexico data is publicly available at http://www.dgis.salud.gob.mx/contenidos/sinais/s_saeh.html. The USA data is publicly available, on signing a data use agreement, at https://www.distributor.hcup-us.ahrq.gov/ .

# Field-specific reporting

Please select the one below that is the best fit for your research. If you are not sure, read the appropriate sections before making your selection.

☐ Life sciences ☐ Behavioural & social sciences ☒ Ecological, evolutionary & environmental sciences

# Ecological, evolutionary & environmental sciences study design

All studies must disclose on these points even when the disclosure is negative.

| | |
|---|---|
| Study description | We use a dataset of RSV cases at the county-level in the USA and bronchiolitis cases at the state level in Mexico to estimate the climate drivers of RSV transmission. We then simulate future epidemics using climate trajectories derived from climate models. |
| Research sample | The sample is state-level hospitalization data from Mexico and county-level data from available counties in USA. |
| Sampling strategy | USA data: we take all data with at least five years of consistent observations where maximum cases exceeds ten. Mexico data: we take all publicly available data. |
| Data collection | The hospitalization data is collected by governmental organizations (Mexico: Sistema Nacional de Información en Salud, USA: Agency for Healthcare Research and Quality). It is publicly available (http://www.dgis.salud.gob.mx/contenidos/sinais/s_saeh.html, https://www.distributor.hcup-us.ahrq.gov/). |
| Timing and spatial scale | For the USA, we take all time series where at least five years of continuous case data are available. Hospitalization data is available from 1989, however, we remove data before 1996 due to a change in reporting at this time. The longest time series spans 1997-2011. For Mexico, the data available spans 2000-2014. USA data contains weekly observations, Mexico data is available daily but is summed to the week level. |
| Data exclusions | We exclude data from locations without continuous observations or with less than five years of case data. In the USA we remove data from counties with very sparse observations (less than 10). |
| Reproducibility | N/A |
| Randomization | N/A |
| Blinding | N/A |

Did the study involve field work? ☐ Yes ☒ No

# Reporting for specific materials, systems and methods

We require information from authors about some types of materials, experimental systems and methods used in many studies. Here, indicate whether each material, system or method listed is relevant to your study. If you are not sure if a list item applies to your research, read the appropriate section before selecting a response.

## Materials & experimental systems

| n/a | Involved in the study |
|---|---|
| ☒ | ☐ Antibodies |
| ☒ | ☐ Eukaryotic cell lines |
| ☒ | ☐ Palaeontology |
| ☒ | ☐ Animals and other organisms |
| ☒ | ☐ Human research participants |
| ☒ | ☐ Clinical data |

## Methods

| n/a | Involved in the study |
|---|---|
| ☒ | ☐ ChIP-seq |
| ☒ | ☐ Flow cytometry |
| ☒ | ☐ MRI-based neuroimaging |
